# CD45RA^+^CCR7^−^ CD8 T cells lacking co-stimulatory receptors demonstrate enhanced frequency in peripheral blood of NSCLC patients responding to nivolumab

**DOI:** 10.1186/s40425-019-0608-y

**Published:** 2019-06-08

**Authors:** Andre Kunert, Edwin A. Basak, Daan P. Hurkmans, Hayri E. Balcioglu, Yarne Klaver, Mandy van Brakel, Astrid A. M. Oostvogels, Cor H. J. Lamers, Sander Bins, Stijn L. W. Koolen, Astrid A. M. van der Veldt, Stefan Sleijfer, Ron H. J. Mathijssen, Joachim G. J. V. Aerts, Reno Debets

**Affiliations:** 1 Laboratory of Tumor Immunology, Department of Medical Oncology, Erasmus MC Cancer Institute, Office Be 430b, Wytemaweg 80, 3015 CN Rotterdam, The Netherlands; 2 Laboratory of Translational Pharmacology, Erasmus MC Cancer Institute, Rotterdam, The Netherlands; 3 Department of Medical Oncology, Erasmus MC Cancer Institute, Rotterdam, The Netherlands; 4 Department of Pulmonary Diseases, Erasmus MC Cancer Institute, Rotterdam, The Netherlands

**Keywords:** NSCLC, Nivolumab, T cells, Biomarkers, Co-stimulatory receptors

## Abstract

**Background:**

Checkpoint inhibitors have become standard care of treatment for non-small cell lung cancer (NSCLC), yet only a limited fraction of patients experiences durable clinical benefit, highlighting the need for markers to stratify patient populations.

**Methods:**

To prospectively identify patients showing response to therapy, we have stained peripheral blood samples of NSCLC patients treated with 2nd line nivolumab (*n* = 71), as well as healthy controls, with multiplex flow cytometry. By doing so, we enumerated 18 immune cell subsets and assessed expression for 28 T cell markers, which was followed by dimensionality reduction as well as rationale-based analyses.

**Results:**

In patients with a partial response (PR), representing best overall response (BOR) according to RECIST v1.1, the number of CD8 T cells at baseline and during treatment is similar to those of healthy controls, but 2-fold higher than in patients with progressive and stable disease (PD and SD). CD8 T cell populations in PR patients show enhanced frequencies of T effector memory re-expressing CD45RA (TEMRA) cells, as well as T cells that express markers of terminal differentiation (CD95+) and egression from tumor tissue (CD69-). In PR patients, the fraction of CD8 T cells that lacks co-stimulatory receptors (CD28, ICOS, CD40L, 4-1BB, OX40) correlates significantly with the total numbers and differentiated phenotype of CD8 T cells.

**Conclusions:**

This study demonstrates that high numbers of peripheral CD8 T cells expressing differentiation markers and lacking co-stimulatory receptors at baseline are associated with response to nivolumab in NSCLC patients.

**Electronic supplementary material:**

The online version of this article (10.1186/s40425-019-0608-y) contains supplementary material, which is available to authorized users.

## Introduction

The onset of T cell activation and differentiation, generally a consequence of the T cell receptor (TCR) recognizing its cognate antigen, is usually accompanied by up-regulated expression of co-inhibitory receptors such as programmed-death 1 (PD-1), proving a negative feedback mechanism to keep T cell activity ‘in check’ [1, 2]. Many types of cancer exploit this adaptive immunity and demonstrate high expression levels of co-inhibitory ligands such as PD-L1 to resist anti-tumor T cell responses. Clinical use of nivolumab, a monoclonal antibody targeting PD-1, showed promising results in metastatic melanoma [3], NSCLC [4, 5] as well as various other types of cancer [6]. Collectively, however, study results reveal that only a limited subset of patients experiences durable clinical benefit [7]. This highlights the need for markers that would identify patients prone to responding to treatment at an early time point and select these patients for extended treatment, thereby avoiding further exposure of patients with limited benefit to a potentially toxic and costly treatment.

Initial searches for predictive markers focused on the expression of PD-L1 [8, 9], but despite FDA approval for patient stratification based on PD-L1 expression in primary tumor tissue of NSCLC patients, interpretation of such immune stainings with respect to cell type and optimal cut off percentage remains challenging [10, 11]. Similarly, investigations assessing tumor mutational burden (TMB), mismatch repair deficiency (dMMR) and microsatellite instability (MSI) reveal that a high score on each of these markers correlates with enhanced responsiveness to anti-PD-1 therapy [12, 13], but on their own these markers may not be sufficiently discriminative to predict clinical response. Also, CD8 T cell density within tumor biopsies has been shown to predict anti-PD-1 response in patients diagnosed with advanced melanoma [14]. Interestingly, local CD8 T cell immunity is affected by escape mechanisms [15], and profiles based on multiple immune parameters, such as the presence of effector cells, MHC molecules, suppressor cells, as well as immune and metabolic checkpoints provide predictive value exceeding that of single markers such as PD-L1 or mutational load [16–18]. However, limited availability of biopsy tissue and its invasiveness, especially in case of visceral tumors, often limits in situ determination of such markers. Multi-parameter analysis of immune cell subsets in blood is an easily employable screening method anticipated to reveal surrogate markers for clinical responses. Indeed, the absolute number of lymphocytes in blood samples correlates with clinical outcome in melanoma patients treated with ipilimumab, a monoclonal antibody targeting the co-inhibitory receptor CTLA-4 [19]. And more recently, Ki67 expression in a subset of PD-1^+^ CD8^+^ T cells has been reported as a measure of effector T cell invigoration in patients with advanced melanoma and NSCLC who were treated with antibodies targeting the PD-1/PD-L1 axis [20–22].

In the current study, we have enumerated 18 immune cell populations and performed both cluster and selected analyses to assess differential frequencies of multiple T cell subsets using 28 markers of T cell activation, maturation, co-signaling and chemotaxis in NSCLC patients treated with 2nd line nivolumab in order to obtain prospective immune markers identifying those patients showing a clear response to therapy.

## Materials and methods

### Study design

The MULTOMAB study (local ethics board study number MEC16–011) was originally designed by the Laboratory of Translational Pharmacology, Dept Medical Oncology at the Erasmus MC Cancer Institute (PIs: R. Mathijssen; J. Aerts and R. Debets). Patients asked to participate in the reported analysis are suffering from NSCLC and receiving treatment in the form of nivolumab (BMS936558, Opdivo®). Written informed consent was obtained from all participants prior to inclusion into the study.

### Patients and collection of specimens

Data was prospectively collected from NSCLC patients treated with 3 mg/kg of nivolumab (intravenously every 2 weeks) between May 5th 2016 and November 1st 2017, with a minimum follow-up of three months. Patient characteristics are provided in Additional file 1: Table S1. Blood was drawn at 3 time points (pre-treatment (“baseline”) and prior to 2nd and 3rd administration of nivolumab (visits (V) 1 and 2). For an overview of patient treatment and sample acquisition, see Additional file 1: Figure S1. Freshly obtained, whole blood was used to enumerate immune cell populations, whereas PBMCs were isolated using ficoll gradient and stored using standard protocols and thawed at later time points to assess frequencies of T cell subsets. Healthy control samples were obtained from 15 donors that were matched with patients for age and gender-distribution (median age: 65 years (60–69); 6 female (40%) and 9 male (60%) donors) (Sanquin, Amsterdam, The Netherlands).

### Assessment of tumor volume and clinical response

Baseline tumor burden was defined as the sum of the longest diameter of all target lesions. Best overall response (BOR) was assessed according to RECIST v1.1. Partial response (PR) was defined as a minimal decrease of 30% in the sum of diameters of the target lesions, taking as reference the sum of diameters at baseline, while progressive disease (PD) was defined as a minimal increase of 20% in the sum of diameters of the target lesions, taking as reference the smallest sum of diameters while on study and a minimal absolute increase of 5 mm. Stable disease (SD) was defined as insufficient change in tumor sizes to qualify for either PR or PD and if duration of SD was 90 days or more. Patients with non-measurable lesions were excluded from analysis. All three BOR response groups displayed similar medians and ranges with regard to age, sex and histology of primary lung tumor.

### Flow cytometry

Whole blood was stained and after lysis of red blood cells analyzed by multi-color FCM on a BD 3-laser Celesta flow cytometer using FACSDIVA 8.x software. Absolute cell counts were determined using Flow-Count Fluorospheres (Beckman Coulter). Cryopreserved PBMC samples were thawed and stained with a master mix of antibodies. Please refer to Additional file 1: Table S2 for an overview of staining panels and utilized markers; all panels were optimized, compensated using Fluorescence minus one (FMO) controls and measurements were corrected for background fluorescence; a detailed list of antibodies is available upon request. Data were gated and analyzed using FlowJo software (Tree Star). Please refer to Additional file 1: Table S3 for an overview of our data analysis work scheme, in which dimensionality reduction analysis (tSNE, see below) preceded two-dimension (2D) analysis of selected markers. The latter analysis of large datasets was conducted using R.

### T-distributed stochastic neighbor embedding (tSNE) analysis

tSNE analysis was performed using the Cytosplore software, with an interactive graphical user interface. CD8 T cell populations were extracted as individual .fcs files and imported into Cytosplore [23], where they were down-sampled to at most 1000 cells per sample, and tSNE analysis was performed on these 211,000 ± 6000 data points (cells from 71 patients, 3 time points each). Clustering was carried out with gradients of density plots, where first a threshold (sigma) of 26 was used, which provided 22 ± 8 clusters per combination of markers (see Additional file 1: Table S2, panels 2–6). This threshold was iteratively increased to a lower number of clusters in such a way that differential marker intensities were not compromised, providing a total of 12 ± 4 clusters per combination of markers. A total of 58 clusters was identified across all markers. The marker intensity profiles and contributions of individual BORs in these clusters were extracted from Cytosplore to excel sheets (Microsoft) for visualization.

### Statistics

tSNE-identified clusters were tested for differential abundance among BOR groups and time points using the Student’s T-test of the scipy stats package in python, while 2D analysis of selected markers was conducted using the Kruskal Wallis test. Descriptive statistics included median, standard deviation and range for continuous variables. For comparison of median differences between individual BOR groups the Mann–Whitney U test was used. For normally distributed data, significant changes of median cell numbers or frequencies within BOR groups over time were determined using two-sided, paired Student’s T-test. Correlations between continuous variables were determined by Pearson’s r coefficient. Differences were considered significant with a *p*-value below 0.05.

### Data reporting

In this discovery study, experiments were not randomized and the investigators were not blinded to patient sample allocation during experiments and outcome assessment.

## Results

### NSCLC patients with PR to nivolumab harbor normal, non-decreased numbers of CD8 T cell numbers in blood in contrast to PD and SD

**Fig. 1:**
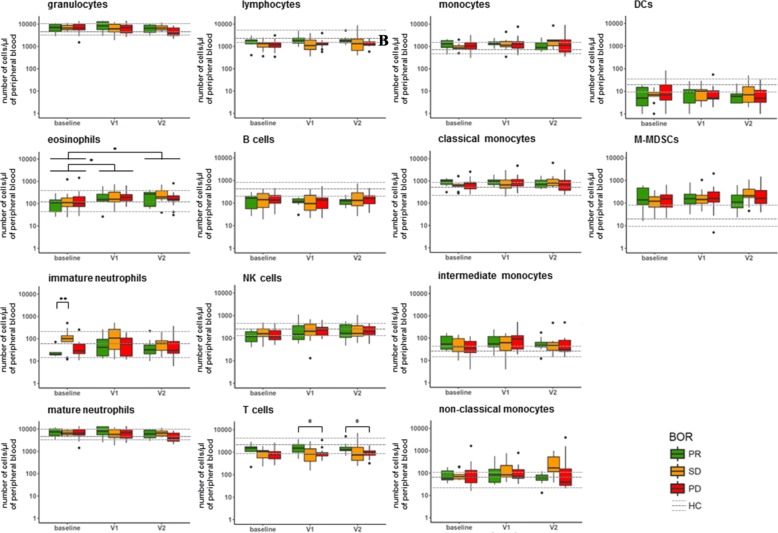
Nivolumab treatment does not result in changed numbers of peripheral immune cell populations, except eosinophils and T cells. Blood samples taken from patients at baseline, V1 and V2 were stained, ery-lysed and subsequently analyzed by multi-color FCM. Immune cell populations that were enumerated and markers used are listed in Additional file 1: Table S2, panel 1. Median numbers of immune cell populations of healthy controls are indicated by a dark grey, dotted line, and upper and lower quartile ranges are indicated by light grey dotted lines. Statistically significant differences between BOR groups were determined using Mann–Whitney U test. * *p* < 0.05; ** *p* < 0.01. BOR = best overall response, PR = partial response, SD = stable disease, PD = progressive disease, HC = healthy control

**Fig. 2:**
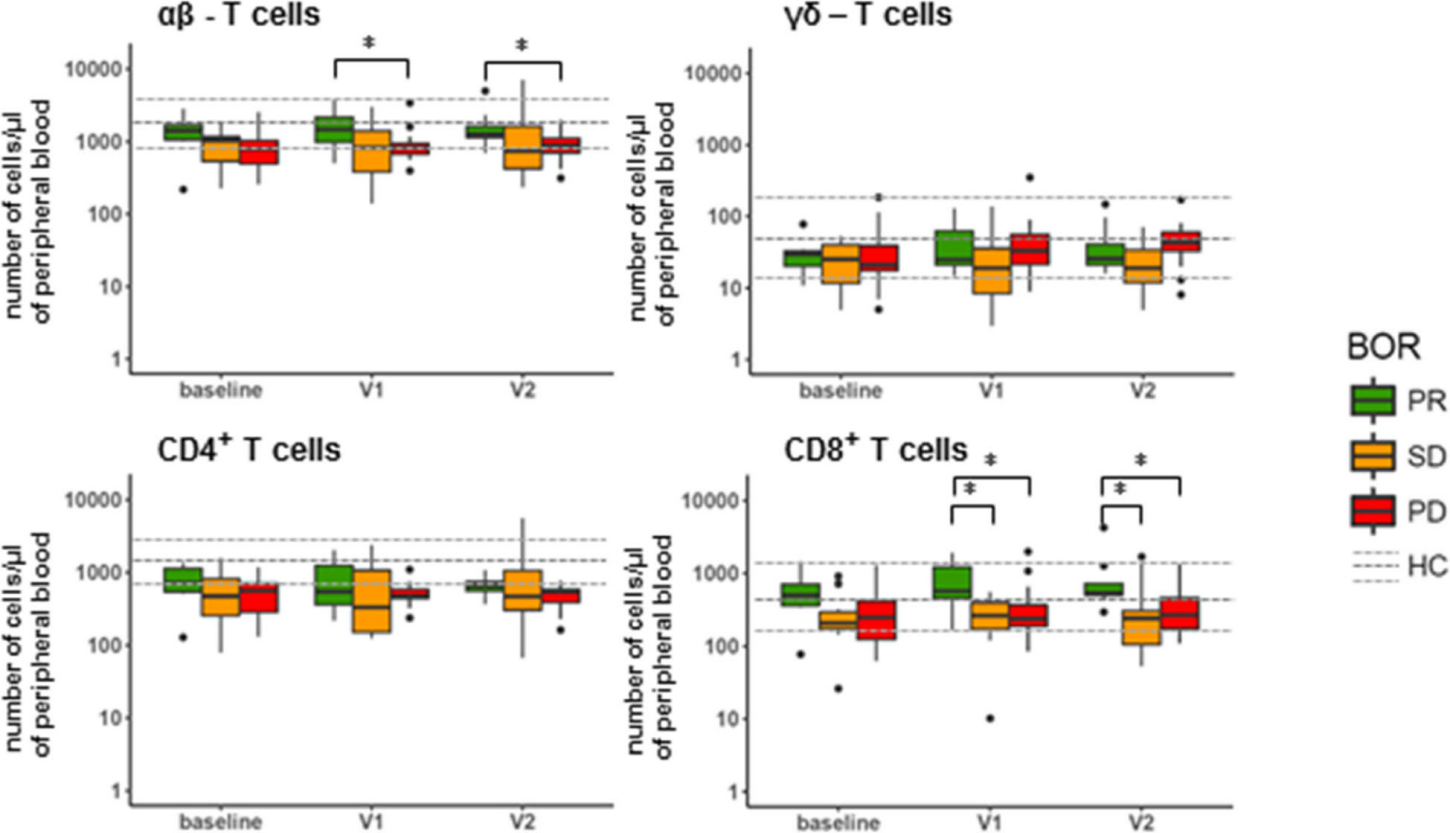
Patients responding to nivolumab show high numbers of CD8 T cells. Graphs show numbers of αβ and γδ T cells in peripheral blood and the respective CD4^+^ and CD8^+^ subsets of αβ T cells. See legend to Fig. 1 for details, abbreviations and statistical testing

Availability of freshly obtained, peripheral blood of 32 of the 71 NSCLC patients enrolled in this study allowed us to conduct enumeration of 18 major immune cell populations prior to and following nivolumab treatment (for treatment and patient details, please refer to Additional file 1: Figure S1 and Additional file 1: Table S1). Patients were assessed for their best overall response (BOR) according to RECIST v1.1 within a follow-up time of at least 90 days (except for patients experiencing progressive disease (PD) within that timeframe) and categorized into patients with partial response (PR; *n* = 7), stable disease (SD; *n* = 10) or PD (*n* = 15). For reference purposes, the same immune cell populations were enumerated in a control group of age and gender-matched healthy individuals (n = 15). Figure 1 depicts the numbers of immune cells detected per μl of peripheral blood at baseline, after the 1st treatment cycle (2 weeks after baseline, visit (V)1) and 2nd treatment cycle (4 weeks after baseline, V2). Numbers remained unchanged after onset of therapy for the majority of immune cell populations, except for eosinophils, which increased in numbers, independent of BOR, and T cells, which differed significantly between PR and PD patients after onset of therapy (see below). When compared to healthy reference values at baseline (see Additional file 1: Figure S2), numbers of granulocytic and myeloid cell populations were enhanced in all BOR groups, i.e., mature neutrophils, monocytes and M-MDSCs, while numbers of lymphocytes (i.e. B and NK cells), were decreased. At baseline, SD patients displayed an enhanced number of immature neutrophils compared to PR patients, who in turn displayed significantly lowered numbers of these cells compared to healthy controls samples. On the other hand, compared to these healthy reference values, median numbers of T cells at baseline were significantly decreased only in PD and SD, but not in PR patients (see Additional file 1: Figure S2). When assessing the major T cell populations, we observed that αβ-T cells, but in particular their CD8-positive subset represented the T cell population that attributed to the difference among the BOR groups (Fig. 2). In example, at baseline we measured a median of 500 CD8 T cells/μl (range: 80–1450) in PR patients, while in SD and PD patients we measured 210 CD8 T cells/μl (30–900) (*p* = 0.061) and 250 CD8 T cells/μl (60–1250) (*p* = 0.057), respectively. This difference increased after onset of therapy. Namely, at time point V1 we measured a median of 560 CD8 T cells/μl (170–1900) in PR patients, while PD and SD patients showed medians of 220 CD8 T cells/μl (90–1070) (*p* = 0.032) and 230 CD8 T cells/μl (10–550) (*p* = 0.01), respectively. Neither γδ-T cells, nor the CD4-positive αβ-T cell subset displayed significant differences between the three BOR groups.

### PR patients show enriched frequencies of CD8 T cells with a phenotype that corresponds to enhanced T cell differentiation

**Fig. 3:**
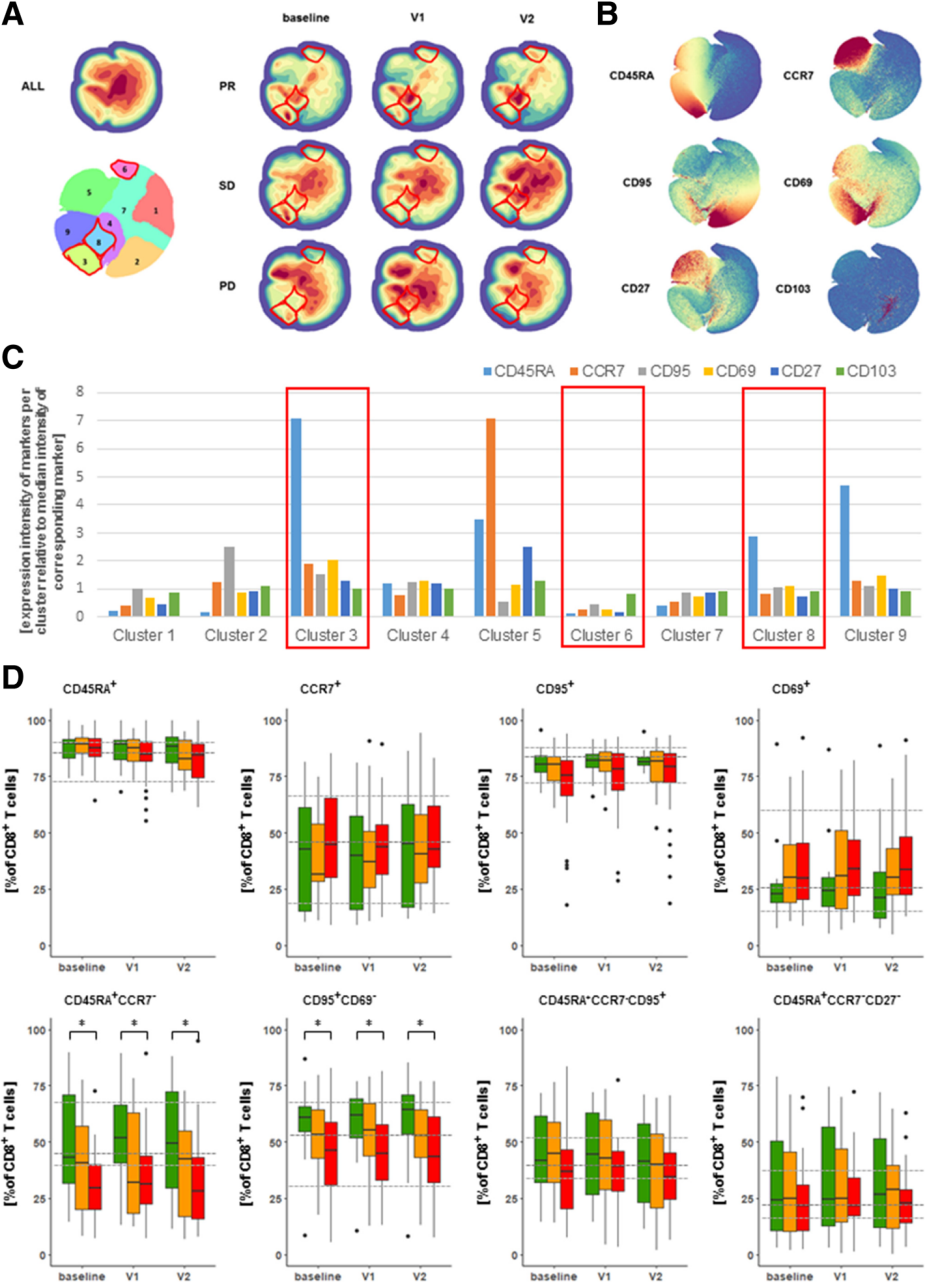
Patients with PR show enhanced frequencies of CD8 T cells with CD45RA^+^CCR7^−^ and CD95^+^CD69^−^ phenotypes. (**a**) Density plots of all data points (ALL: cells from 71 patients, 3 time points each) and split up according to BOR and time points. Plot with 9 clusters (lower left) is the result of gradients of density plots and iterative testing (see Materials and Methods for details). Individual clusters were assessed for significant differences between BOR groups and time points, and highlighted by red lines (see also Additional file 1: Figure S3A). (**b**) Density plots of individual markers and (**c**) expressions of markers within individual clusters according to relative intensities; clusters showing different abundance (from panel A) are highlighted by red rectangles. (**d**) Frequencies of CD8 T cells positive for single markers or combinations of two markers. Markers used are listed in Additional file 1: Table S2, panel 2. Statistically significant differences between BOR groups and time points were determined using Mann–Whitney U test. * *p* < 0.05

As numbers of CD8 T cells differed between patients in the different BOR groups, we further investigated their particular subsets in more detail. To this end, we stained peripheral blood mononuclear cell (PBMC) samples of a total of 71 NSCLC patients (PR: *n* = 14; SD: *n* = 25; PD: *n* = 32) for 28 markers (Additional file 1: Tables S2 and S3), followed by dimensionality reduction as well as rationale-based analysis to identify (combinations of) markers from each of our flow cytometry panels that would reveal significant differences between BOR groups and time points within the CD8 T cell subset (identical analysis was conducted in CD4 T cells; data not shown). Starting with T cell maturation markers, and taking into account all patients and time points, density plots revealed 9 distinct clusters of which 5 were differently abundant between BOR groups and time points (Fig. 3a). In example, clusters 3 and 8 displayed higher densities in PR patients when compared to PD patients (significantly different clusters are highlighted by red lines in Fig. 3a; see also Additional file 1: Figure S3A). Zooming in on density plots of markers (Fig. 3b) and expression intensities of those markers within individual clusters (Fig. 3c), we observed that differences in above-mentioned clusters were mostly attributed to CD45RA, CCR7, CD95 and CD69. Instructed by these cluster analyses as well as reported combinations of T cell maturation markers, we observed that frequencies of CD8 T cells expressing single maturation markers were not different (Fig. 3d, upper row), whereas frequencies of CD8 T cells expressing CD45RA and lacking CCR7 as well as those expressing CD95 and lacking CD69 were different among BOR groups (Fig. 3D, lower row). In fact, PR patients showed an enhanced frequency of CD45RA^+^CCR7^−^ CD8 T cells at baseline (median: 43.1%) when compared to PD patients (29.7%). Moreover, PR but not PD patients showed a trend of increased frequency of CD45RA^+^CCR7^−^ CD8 T cells during nivolumab treatment (52 and 31% at V1 for PR and PD, respectively). Additionally, PR, SD and PD patients showed 60, 53 and 46% of CD95^+^CD69^−^ CD8 T cells at baseline, respectively (Fig. 3d; *p* = 0.033 PR v. PD). Furthermore, CD4 T cells displayed no differences between BOR groups with regard to maturation and differentiation markers (data not shown).

When assessing CD8 T cell frequencies according to markers of proliferation and regulatory T cells in an identical manner (Additional file 1: Figures S3B and S4), we identified clusters with significant, albeit low intensity differences between BOR groups. However, neither frequencies of CD8 T cells expressing individual markers nor those expressing combinations of markers, such as CD25 and FOXP3, were differently present among BOR groups. Notably, frequency of CD4 regulatory T cells showed no difference between BOR groups (data not shown). Frequencies of CD8 T cells expressing the proliferation marker Ki67 either as a single marker or in combination with PD-1 did not show significant differences between BOR groups either. It is noteworthy, however, that we did observe a significant increase in frequency of Ki67^+^ CD8 T cells expressing PD-1 after onset of therapy in all BOR groups (Additional file 1: Figure S5) and that there was a positive correlation between frequency of Ki67^+^ within PD1^+^CD8 T cells and pre-treatment tumor volume of target lesions in NSCLC patient. This correlation, however, was not predictive of response to therapy.

### PR patients show decreased frequencies of CD28^+^CD40L^+^ and CD28^+^ICOS^+^ CD8 T cells

**Fig. 4:**
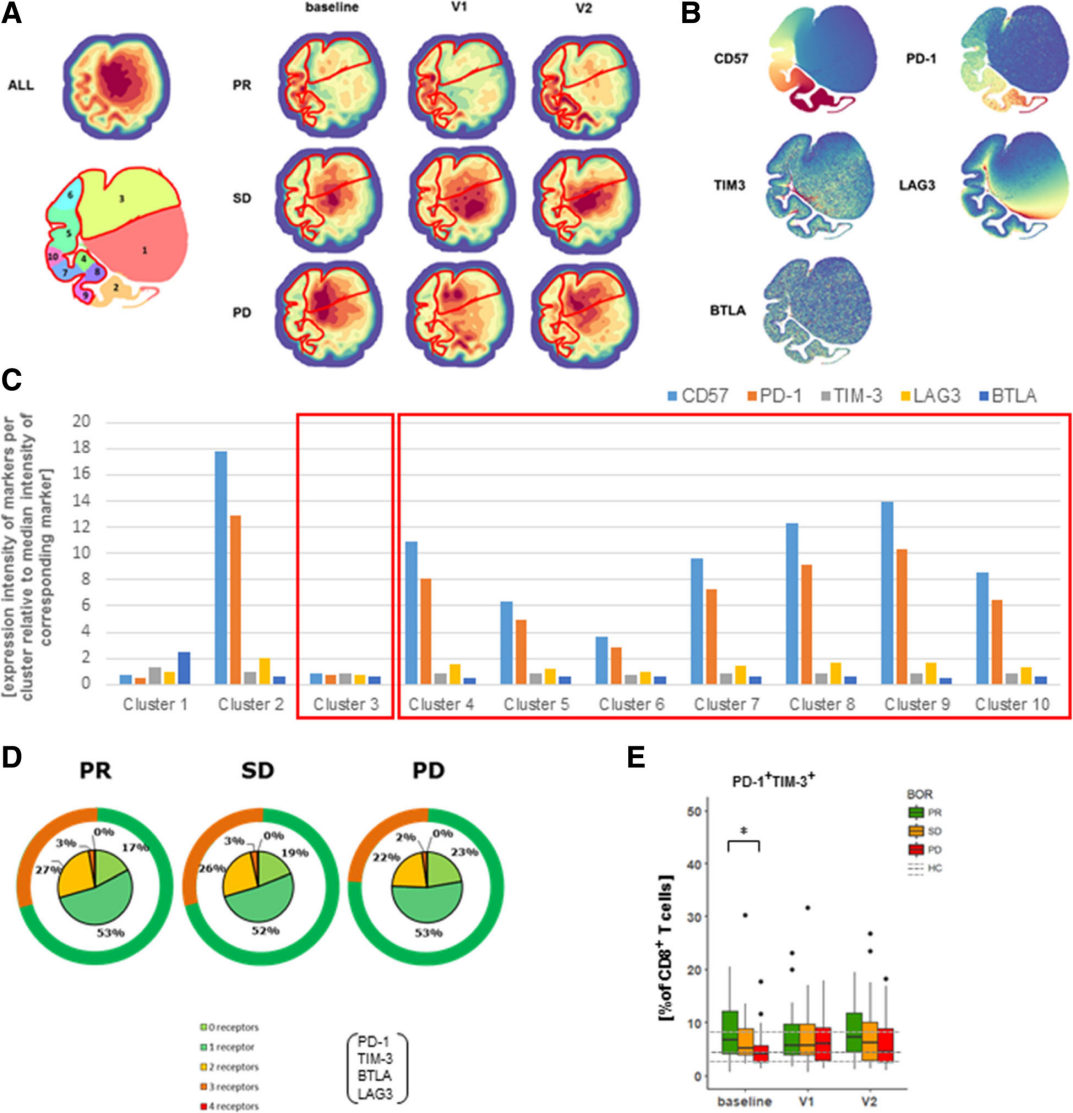
Patients with PR display enhanced frequency of PD-1^+^TIM3^+^ CD8 T cells at baseline. (**a**) Density plots of all data points (ALL: cells from 71 patients, 3 time points each) and split up according to BOR and time points. Plot with 10 clusters (lower left) is the result of gradients of density plots and iterative testing (see Materials and Methods for details). Individual clusters were assessed for significant differences between BOR groups and time points, and highlighted by red lines (see also Additional file 1: Figure S3C). (**b**) Density plots of individual markers and (**c**) expressions of markers within individual clusters according to relative intensities; clusters showing different abundance (from panel A) are highlighted by red rectangles. (**d**) Sum of different types of co-inhibitory receptors that are expressed by CD8 T cells (excluding CD57) at baseline. Green circles visualize fraction of CD8 T cells expressing 0 or 1 type of co-inhibitory receptors. (**e**) Frequencies of CD8 T cells positive for single markers or combinations of two markers showing significant differences. Markers used are listed in Additional file 1: Table S2, panel 4. Statistically significant differences between BOR groups and time points were determined using Mann–Whitney U test. * *p* < 0.05

**Fig. 5:**
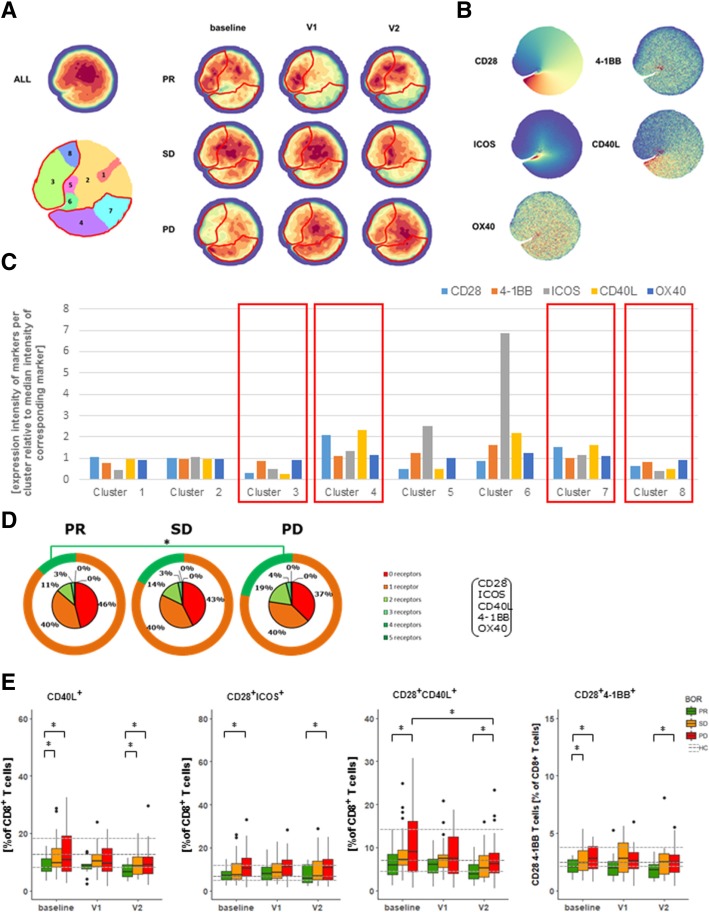
Patients with PR display reduced frequencies of CD8 T cells co-expressing CD28 and CD40L or CD28 and ICOS. (**a**) Density plots of all data points (ALL: cells from 71 patients, 3 time points each) and split up according to BOR and time points. Plot with 8 clusters (lower left) is the result of gradients of density plots and iterative testing (see Materials and Methods for details). Individual clusters were assessed for significant differences between BOR groups and time points, and highlighted by red lines (see also Additional file 1: Figure S3D). (**b**) Density plots of individual markers and (**c**) expressions of markers within individual clusters according to relative intensities; clusters showing different abundance (from panel A) are highlighted by red rectangles. (**d**) Sum of different types of co-stimulatory receptors that are expressed by CD8 T cells at baseline. Orange circles visualize fraction of CD8 T cells expressing 0 or 1 type of co-stimulatory receptors. (**e**) Frequencies of CD8 T cells positive for single markers or combinations of two markers with significant differences. Markers used are listed in Additional file 1: Table S2, panel 5. Statistically significant differences between BOR groups and time points were determined using Mann–Whitney U test. * *p* < 0.05

When looking into expression of co-inhibitory receptors, we identified several density clusters that showed significant differences between BOR groups and time points (Fig. 4a and Additional file 1: Figure S3C). Differences in the majority of these clusters were attributed to CD57 and PD-1 (Fig. 4b and c). In addition to these findings, we have assessed the sum of different co-inhibitory receptors expressed by CD8 T cells (i.e., BTLA, PD-1, TIM3, LAG3), and noted that PR patients have a trend of expressing higher frequencies of CD8 T cells with 2 or more different co-inhibitory receptors when compared to PD patients at baseline (Fig. 4d). Instructed by these analyses, we observed that frequencies of CD8 T cells expressing a single type of co-inhibitory receptors were not different, whereas frequencies of CD8 T cells co-expressing PD-1 and TIM3 were more frequent in PR patients when compared to PD patients at baseline (Fig. 4e). This finding extends the observation that the frequency of highly differentiated CD8 T cells is enhanced in PR patients. Using our panel of co-stimulatory receptors, we again identified density clusters that are differentially abundant among BOR groups and time points (Fig. 5a, Additional file 1: Figure S3D). Interestingly, clusters that were more abundant in PR patients were marked by a decreased presence of CD28, ICOS and CD40L (clusters 3 and 8 in Fig. 5b and c), whereas clusters that were more abundant in PD patients were marked by an increased presence of CD28 and CD40L (clusters 4 and 7). When assessing the sum of different receptors expressed by CD8 T cells, we noted that PR patients were marked by a higher frequency of CD8 T cells devoid of all five co-stimulatory receptors (i.e., CD28, ICOS, CD40L, 4-1BB and OX40). PR patients had lower frequencies of CD8 T cells with 2 or more different co-stimulatory receptors when compared to PD patients at baseline (Fig. 5d). Frequencies of CD8 T cells expressing a single type of co-stimulatory receptors, except a lower frequency of CD40L^+^ CD8 T cells, were not different among BOR groups nor time points (Fig. 4e). In contrast, analysis of frequencies of CD8 T cells expressing 2 co-stimulatory receptors revealed that T cells expressing CD28 combined with another receptor, particularly CD40L or ICOS, were lowest in PR and significantly higher in PD patients (Fig. 5e).

### In PR patients, the CD8 T cell differentiation phenotype coincides with a complete lack of co-stimulatory receptors

**Fig. 6:**
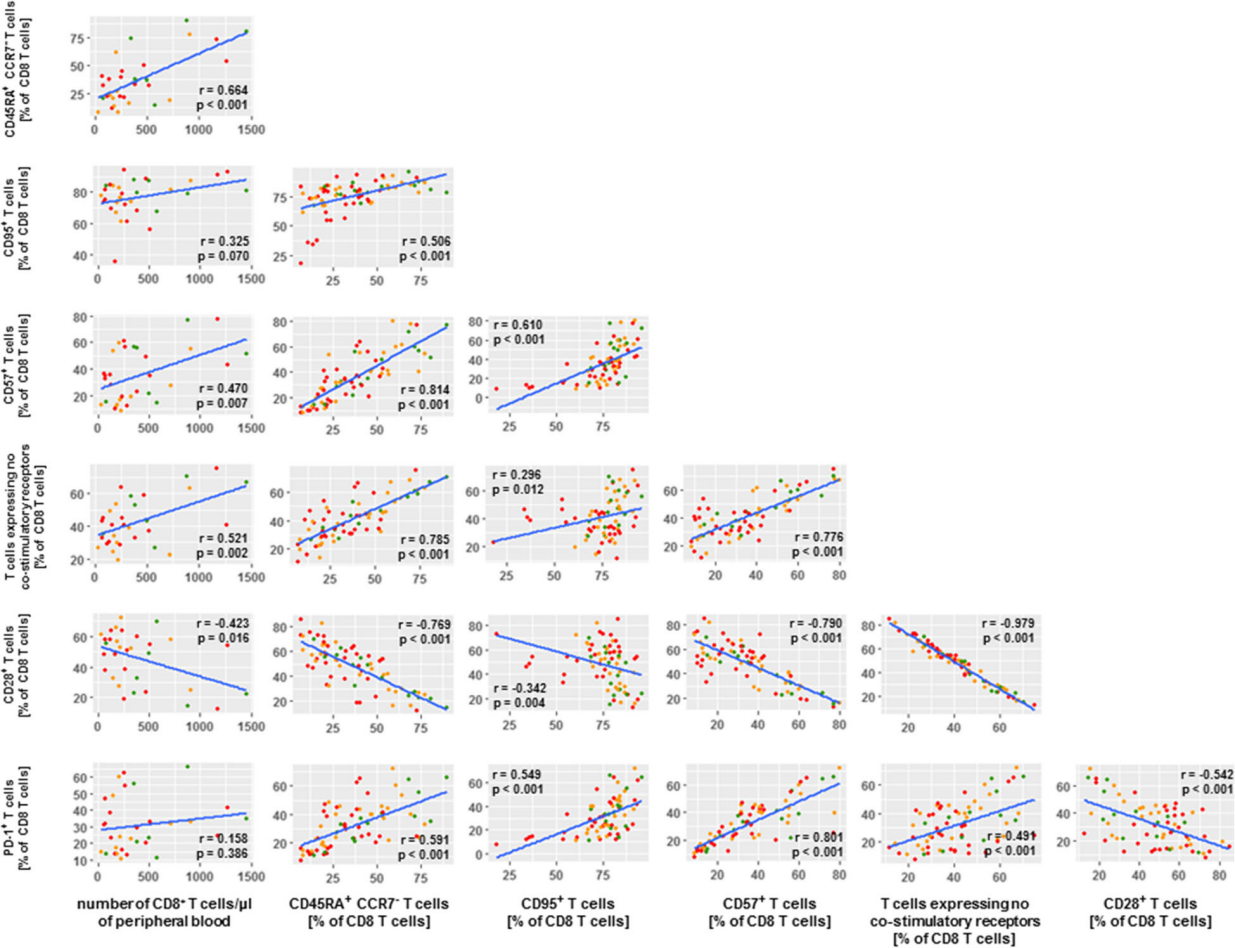
Number of CD8 T cells in PR patients correlate with CD8 T cell maturation phenotypes. Correlation matrix depicts CD8 T cell phenotypes that were selected according to statistically significant differences between BOR groups (*p* values < 0.001) as well as extent of correlations with number of CD8 T cells and frequency of T cell phenotypes (r values < − 0.5 and > 0.5). Correlations were statistically assessed via Spearman’s test

To study whether and how the differential numbers of CD8 T cells as well as the differential frequencies of defined CD8 T cell phenotypes among BOR groups were inter-related, we conducted extensive correlation studies with all immune markers measured in this study. Figure 6 displays the resulting matrix of immune parameters with the highest correlations (r values < − 0.5 or > 0.5 and *p* values < 0.001) with number of CD8 T cells and the CD8 phenotypes. Enhanced numbers of CD8 T cells in PR patients relate most clearly to frequencies of CD45RA^+^CCR7^−^ CD8 T cells as well as CD8 T cells with no co-stimulatory receptors. In turn, frequencies of CD45RA^+^CCR7^−^ CD8 T cells predominantly relate to frequencies of CD95^+^ CD8 T cells, CD57^+^ CD8 T cells, PD-1^+^ CD8 T cells and again CD8 T cells with no co-stimulatory receptors.

## Discussion

In this explorative study, we set out to discover potential immune markers in NSCLC patients that correspond with response to nivolumab therapy. The distribution of BOR in this prospective study of 71 patients is reflective of clinical outcome in large clinical trials with NSCLC patients [4, 5] with about 20% of treated patients showing response. Using our prospectively collected cohort of patients, we have enumerated immune cell populations and assessed clusters of T cell markers and frequencies of T cells subsets in blood samples drawn prior to and during therapy, using reference values from age- and gender-matched healthy controls.

Most studies evaluating systemic immune profiles generally rely on frozen PBMC samples, resulting in a bias towards immune cell populations that show high stability throughout the freeze/thaw procedure [24]. To address this issue, we have determined numbers of 18 different immune cell populations in freshly obtained blood. Amongst the significant differences in numbers of major immune cell populations between the three BOR groups, we detected a general increase in numbers of eosinophils during nivolumab therapy. Such an increase in peripheral eosinophils has previously been identified as a prognostic marker for survival in metastatic melanoma patients treated with various types of immune therapy [25]. However, increase in eosinophils was not associated with BOR in our NSCLC cohort as this increase occurred irrespective of BOR. At baseline, only immature neutrophils and T cells, in particularly CD8 T cells, showed differences among BOR groups. The increased number of immature neutrophils in SD patients is interpreted with caution since this finding may have been the result of exclusion of several outliers in this particular BOR group at baseline, part of our downstream analysis, which may have reduced the spread in this immune cell subset. The reduced number of CD8 T cells in SD and PD patients prior to therapy on the other hand shows a relatively low spread and is consistent over time. The latter observation may explain the lack of responsiveness to therapy and is supported by previous findings of reduced numbers of T cells (CD45^+^CD3^+^) during immune checkpoint inhibition [19]. Besides therapy-induced changes, we also observed changed numbers of immune cell populations at baseline when compared with healthy controls. Increased numbers of mature neutrophils and monocytes correspond with an inflamed tumor microenvironment that may drive the proliferation of these cells and their detection in the periphery [26]. Also, our finding of increased numbers of M-MDSCs is in line with multiple reports, and may be of interest since these cells have been described as main suppressors of immune responses [27, 28]. The role of activated NK cells (expressing MIP-1β and CD69) in the context of anti PD-1 therapy of melanoma patients has recently been highlighted by Hodi and colleagues [29]. These authors observed increased frequencies of these cells as well as NK cells in patients showing response to therapy. Important to note that numbers of neutrophils, M-MDSCs, B or NK cells, neither by themselves nor in combination with other immune cell populations, did correlate with BOR in the present study, indicating that immune response in NSCLC patients may be mostly driven by T cells, rather than NK, B or other effector cells.

To follow-up on the different CD8 T cell numbers, we conducted a dimensionality reduction as well as 2D analyses to identify marker combinations and T cell subsets. Notably, we observed that reduced numbers of CD8 T cells in SD and PD patients were not due to changed frequencies of CD8 regulatory T cells nor a general lack of T cell proliferation (Additional file 1: Figure S4). Although the presence of CD4 Treg cells within the tumor microenvironment has been described as a potential driver of tumor immune escape (reviewed in [30]), peripheral frequencies of this subset may not be sufficiently reflective of local conditions. An increase in the frequency of PD-1^+^ CD8 T cells and an enhanced frequency of PD-1^+^ CD8 T cells that express Ki67 has previously been observed in NSCLC patients undergoing anti-PD-1/anti-PD-L1 therapy [20, 31]. Similar to this study, we found an increase in PD-1^+^ CD8 T cells expressing Ki67, yet no correlation between their frequencies after onset of therapy and the clinical response according to RECIST1.1 (see Additional file 1: Figure S5). Huang and colleagues demonstrated that the ratio between Ki67^+^PD-1^+^ CD8 T cells and pre-therapy tumor burden was indicative of a clinical response of melanoma patients to pembrolizumab [21]. While we observed a similar correlation between 1D tumor measurements and frequencies of Ki67^+^PD-1^+^ CD8 T cells, albeit to a lower degree (see Additional file 1: Figure S5C), we were unable to demonstrate this ratio to be of discriminatory value among BORs in our NSCLC patient cohort. Although we cannot exclude that increased frequencies of Ki67^+^PD-1^+^CD8 T cells depend on tumor type, mutational load and/or certain patient subgroups, our findings do argue that further studies are required to better define how the Ki67 marker relates to clinical response to checkpoint inhibition. When conducting similar tSNE and 2D analysis of chemo-attractant receptors, we observed that the frequency of CD8 T cells expressing such receptors did not yield differences between BOR groups or time points (Additional file 1: Figure S6).

When looking into maturation states of T cells, we detected significantly higher frequencies of CD45RA^+^CCR7^−^ CD8 T cells, a phenotype often related to terminal T cell differentiation [32], in PR patients compared to PD patients at baseline and during treatment. Moreover, in PR patients we observed higher frequencies of CD95^+^CD69^−^ CD8 T cells. While CD95 has been recognized for FAS-mediated apoptosis, there is evidence for FAS-mediated T cell proliferation and differentiation as well [33]. High numbers of CD95^+^ CD8^+^ tumor infiltrating lymphocytes have previously been demonstrated to have predictive value in breast cancer patients [34] and an enhanced frequency of CD95^+^ T cells in blood of stage IV melanoma patients has been reported to associate with clinical response upon anti-PD-1 treatment [22]. CD69 is an early activation marker that shows a rapid and transient upregulated expression upon TCR-mediated activation of CD8 T cells. Additionally, CD69 has been described as a tissue retention marker, indicating that down-regulated expression of CD69 coincides with egress of T cells into the blood flow [35]. Therefore, the observed changes, with respect to both CD45RA^+^CCR7^−^ and CD95^+^CD69^−^ CD8 T cell phenotypes, may be a consequence of local antigen encounter, T cell differentiation, and tissue egression of CD8 T cells in PR patients. Interestingly, these findings are nicely in line with recent observations by Gide and colleagues showing that differentiated effector memory T cells are more abundant in melanoma patients who respond to PD1 and CTLA-4 antibody treatment [36]. Further evidence for enhanced T cell differentiation in PR patients comes from the observation that the frequency of the mentioned phenotypes highly correlates with the frequency of CD8 T cells expressing CD57, another marker of terminal exhaustion upon antigen encounter [37]. Lastly, other CD8 T cell phenotypes that have been reported to relate to late T cell differentiation, such as lack of the co-stimulatory receptor CD28 and co-expression of PD-1 and TIM3, also show enhanced frequencies in PR patients (discussed below). Analysis of co-signaling receptors revealed that clear differences between BOR groups are particularly related to a CD8 T cell subset lacking the co-stimulatory receptors CD28, ICOS, CD40L, 4-1BB and OX40. Interestingly, PR patients show an increased frequency of CD8 T cells lacking co-stimulatory receptors, in particular CD28 and CD40L or CD28 and ICOS. Moreover, the frequency of CD28^+^ CD8 T cells showed a high and inverse correlation with the frequency of CD8 T cells lacking co-stimulatory receptors (Fig. 6). While expression of CD28 is a pre-requisite for proper activation of T cells, the absence of this receptor has been described as part of a negative feedback loop following long-term antigen stimulation [38], and fits the above-described phenotype of antigen-exposed and differentiated CD8 T cells. Further substantiating the premise that a higher frequency of CD8 T cells in PR patients have encountered antigen, is our observation that these patients contain higher frequencies of PD-1^+^TIM3^+^ CD8 T cells at baseline (see Fig. 5c). The combination of these two receptors has been well described as a sign of activation-mediated T cell differentiation and potentially exhaustion [39–41]. Moreover, in patients with squamous cell carcinoma of the head and neck, recent studies showed that PD-1^+^TIM3^+^ CD8 T cells that lack CD28 and CD27 were able to suppress proliferation of autologous peripheral blood T cells ex vivo [42]. Of interest, the presence of intra-tumoral PD-1^+^ CD8 T cells expressing the transcription factor Tcf has been related to tumor control in response to immunotherapy [43, 44] and these T cells may harbor stemness and yield T cells that are more differentiated. Since PD-1 primarily intervenes with CD28 co-signaling, rather than TCR signaling itself [20, 45], we cannot exclude that the frequency of CD28^+^ T cells that co-express Ki67 and PD-1 becomes enhanced upon treatment with checkpoint inhibitor. Along these lines, it is striking that the frequency of CD8 T cells devoid of multiple co-stimulatory receptors is highest in PR patients at baseline and throughout therapy, and correlates with the total number of CD8 T cells as well as frequencies of CD8 T cells showing a CD45RA^+^CCR7^−^ phenotype.

## Conclusions

In conclusion, we found that NSCLC patients with a PR upon treatment with nivolumab demonstrate enhanced numbers of CD8 T cells and a phenotype corresponding with late differentiation at baseline. Collectively, our findings argue that a large fraction of CD8 T cells in PR patients has been exposed to tumor antigen and subsequently matured and egressed into the bloodstream. This enhanced CD8 T cell differentiation was accompanied by a higher frequency of PD-1 and TIM3 and a complete loss of co-stimulatory receptors. We propose that a panel comprising the markers CD45RA, CCR7, CD95, CD69, CD57, PD-1 as well as CD28, CD40L, and ICOS should be validated in larger cohorts of patients and used to develop a model aiding in the identification of NSCLC patients prone to show tumor regression upon anti-PD-1 therapy. While novel approaches are emerging that include assessment of tumor material with regard to T cell exclusion and exhaustion [18], to our knowledge this is the first description of peripheral immune markers able to identify NSCLC patients showing response to nivolumab treatment prior to onset of therapy (see Additional file 1: Figure S7 for a schematic overview of our findings).

## Additional file

Additional file 1: For figure details, please refer to page 1 of additional file 1. **Figure S1.** Timeline of sample acquisition and processing for NSCLC patients treated with nivolumab. **Figure S2**. NSCLC patients demonstrate enhanced numbers of mature neutrophils and M-MDSC in blood, but decreased numbers of dendritic cells, B and NK lymphocytes when compared to healthy subjects. **Figure S3**. Differential abundance of tSNE clusters per panel of markers among BOR groups and time points. **Figure S4**. PR patients display no distinct differences in markers of T cell proliferation nor regulatory T cells. **Figure S5**. Nivolumab-induced increase in frequency of Ki67+ within PD1+CD8 T cells correlates with pre-treatment tumor burden in NSCLC patients, but is not predictive of response to therapy. **Figure S6**. PR patients do not show differential frequencies of CD8 T cells expressing receptors for chemo-attractants. **Figure S7**. Clinical response in NSCLC patients following nivolumab treatment is characterized by high numbers of matured CD8 T cells lacking co-stimulatory receptors. **Table S1**. Patient characteristics. **Table S2**. Multiplex flow cytometry panels. **Table S3**. Analysis work scheme. (PDF 871 kb)

## Abbreviations

BOR: Best overall response
BTLA: B- and T-lymphocyte attenuator
CD: Cluster of differentiation
CTLA-4: Cytotoxic T-lymphocyte–associated antigen 4
dMMR: mismatch repair deficiency
FMO: Fluorescence minus one
ICOS: Inducible T cell co-stimulator
LAG3: Lymphocyte-activation gene 3
MSI: Microsatellite instability
NSCLC: Non-small cell lung cancer
PD: Progressive disease
PD-1: Programmed death 1 receptor
PD-L1: Programmed-death ligand 1
PR: Partial response
SD: Stable disease
TCR: T cell receptor
TIM3: T cell immunoglobulin and mucin-domain containing-3
TMB: Tumor mutational burden TMB
tSNE: t-distributed stochastic neighbor embedding
