# Supplementary material for: CD45RA+CCR7− CD8 T cells lacking co-stimulatory receptors demonstrate enhanced frequency in peripheral blood of NSCLC patients responding to nivolumab
Source: J Immunother Cancer. 2019 Jun 8;7:149. doi: 10.1186/s40425-019-0608-y (PMC6555948; doi:10.1186/s40425-019-0608-y)
Supplement: Supplementary file 1 — For figure details, please refer to page 1 of additional file 1. Figure S1. Timeline of sample acquisition and processing for NSCLC patients treated with nivolumab. Figure S2. NSCLC patients demonstrate enhanced numbers of mature neutrophils and M-MDSC in blood, but decreased numbers of dendritic cells, B and NK lymphocytes when compared to healthy subjects. Figure S3. Differential abundance of tSNE clusters per panel of markers among BOR groups and time points. Figure S4. PR patients display no distinct differences in markers of T cell proliferation nor regulatory T cells. Figure S5. Nivolumab-induced increase in frequency of Ki67+ within PD1+CD8 T cells correlates with pre-treatment tumor burden in NSCLC patients, but is not predictive of response to therapy. Figure S6. PR patients do not show differential frequencies of CD8 T cells expressing receptors for chemo-attractants. Figure S7. Clinical response in NSCLC patients following nivolumab treatment is characterized by high numbers of matured CD8 T cells lacking co-stimulatory receptors. Table S1. Patient characteristics. Table S2. Multiplex flow cytometry panels. Table S3. Analysis work scheme. (PDF 871 kb) [file 40425_2019_608_MOESM1_ESM.pdf]

Additional file 1: Figure details

Figure S1. For immune monitoring, 20 ml of peripheral blood was taken at three time points: prior to therapy (baseline), prior to the second therapy cycle (V1, 2 weeks after baseline) and prior to the third therapy cycle (V2, 4 weeks after baseline). Blood was stained, ery-lysed and 18 different immune cell populations were enumerated by multiplex flow cytometry. PBMCs were isolated and cryopreserved, and stained for 28 markers (for a detailed panel overview, please refer to Table S2) followed by dimensionality reduction as well as rationale-based analyses.

Figure S2. Blood samples were taken from NSCLC patients at baseline as well as from healthy controls (HC), and were stained, ery-lysed and subsequently analyzed by multi-color FCM. See legend to Figure S1 and Table S2 for details. Statistically significant differences between BOR groups and HC were determined using Mann–Whitney U test. * $p < 0.05$, ** $p < 0.01$, *** $p < 0.005$.

Figure S3. Graphs depict % of CD8 T cells (mean + SD) present in tSNE clusters according to BOR groups and time points and categorized by panels of markers (as described in Table S2), namely: (**A**) T cell maturation, (**B**) proliferation/regulatory T cell markers, (**C**) co-inhibitory receptors, (**D**) co-stimulatory receptors and (**E**) chemo-attractant receptors. See legends to Figs. 3, 4, 5, S4 and S6 for details. Red lines indicate differences between BOR groups within the same time point, black lines indicate differences within the same BOR group but between time points. Statistically significant differences were determined using Student’s T test. * $p < 0.05$; ** $p < 0.01$; *** $p < 0.005$.

Figure S4. (**A**) Density plots of all data points (ALL: cells from 71 patients, 3 time points each) and split up according to BOR and time points. Plot with 14 clusters (lower left) is the result of gradients of density plots and iterative testing (see Materials and Methods for details). Individual clusters were assessed for significant differences between BOR groups and time points, and highlighted by red lines (see also Figure S3B). (***B***) Density plots of individual markers and (**C**) expressions of markers within individual clusters according to relative intensities; clusters showing different abundance (from panel A) are highlighted by red rectangles. (**D**) Frequencies of CD8 T cells positive for single markers or combinations of two markers. Markers used are listed in Table S2, panel 3. Statistically significant differences between BOR groups and time points were determined using Mann–Whitney U test. * $p < 0.05$.

Figure S5. Graph in (**A**) shows frequency of Ki67^{+} cells within PD-1^{+}CD8 T cells. Graph (**B**) presents the difference in frequency of Ki67^{+} within PD1^{+}CD8 T cells between BOR groups. Graph in (**C**) depicts correlation between 1D tumor burden at baseline according to radiology (see methods for details) and maximum change in frequency of Ki67^{+} within PD1^{+}CD8 T cells (delta between either V1 or V2 and baseline). Correlations were statistically assessed via Spearman’s test. Statistically significant differences between BOR groups and time points were determined using Mann–Whitney U test and paired Student’s T-test, respectively. * $p < 0.05$, ** $p < 0.01$, *** $p < 0.005$.

Figure S6. (**A**) Density plots of all data points (ALL: cells from 71 patients, 3 time points each) and split up according to BOR and time points. Plot with 17 clusters (lower left) is the result of gradients of density plots and iterative testing (see Materials and Methods for details). Individual clusters were assessed for significant differences between BOR groups and time points, and highlighted by red lines (see also Figure S3E). (**B**) Density plots of individual markers and (**C**) expressions of markers within individual clusters according to relative intensities; clusters showing different abundance (from panel A) are highlighted by red rectangles. (**D**) Frequencies of CD8 T cells positive for single markers or combinations of two markers. Markers used are listed in Table S2, panel 6. Statistically significant differences between BOR groups and time points were determined using Mann–Whitney U test. * $p < 0.05$.

Figure S7. This figure depicts main findings of this study, indicating that patients responding to therapy (PR) prior to start of therapy display higher numbers of peripheral CD8 T cells, with enhanced frequencies of the phenotypes CD45RA^{+}CCR7^{-}, CD95^{+}CD69^{-} and lack of CD28, ICOS, CD40L, 4-1BB and OX40.

Supplementary Figure 1

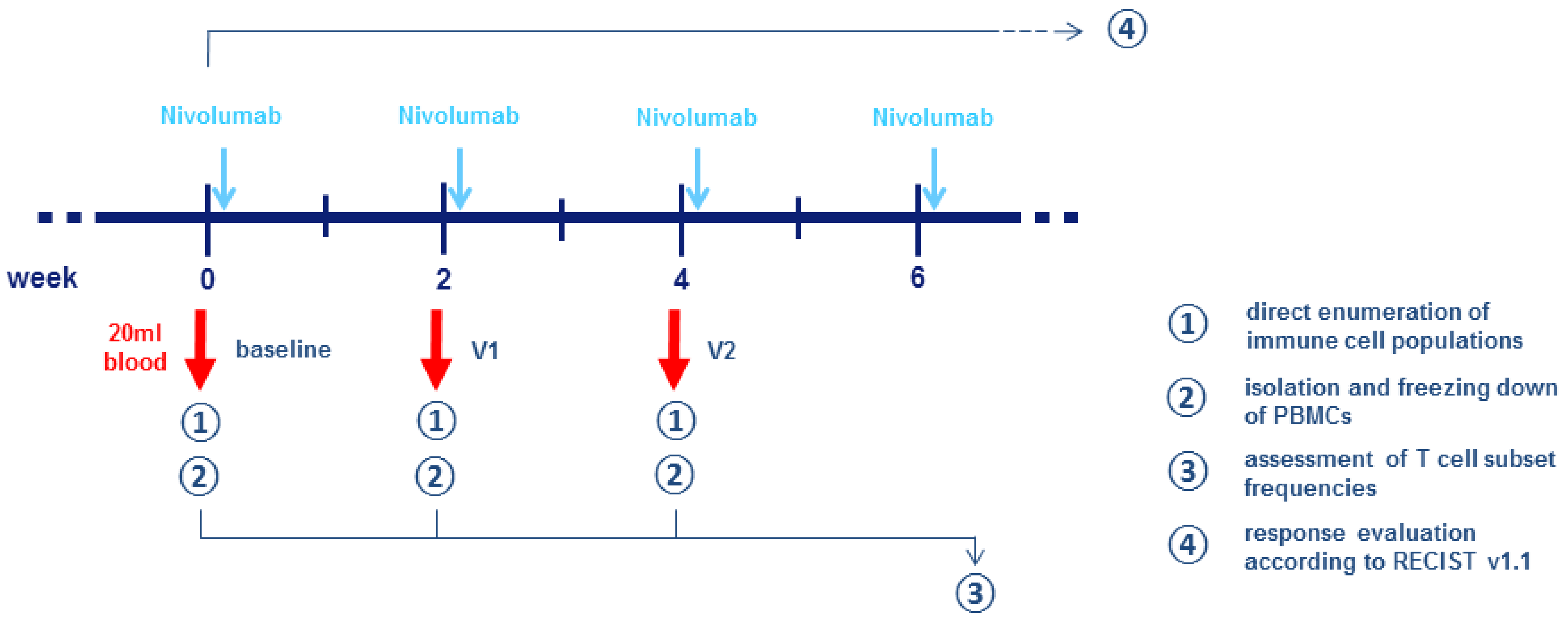

Supplementary Figure 2

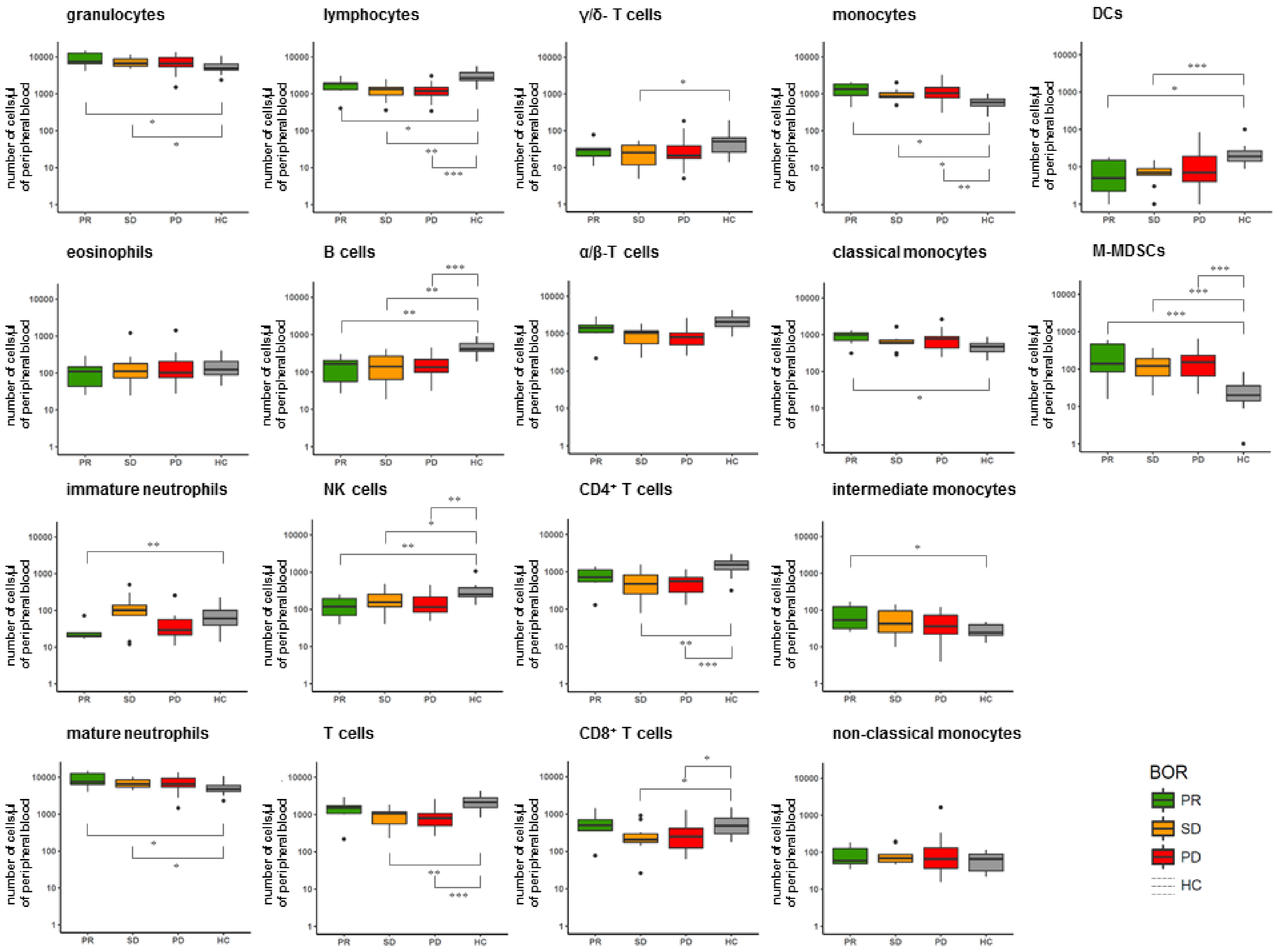

Supplementary Figure 3

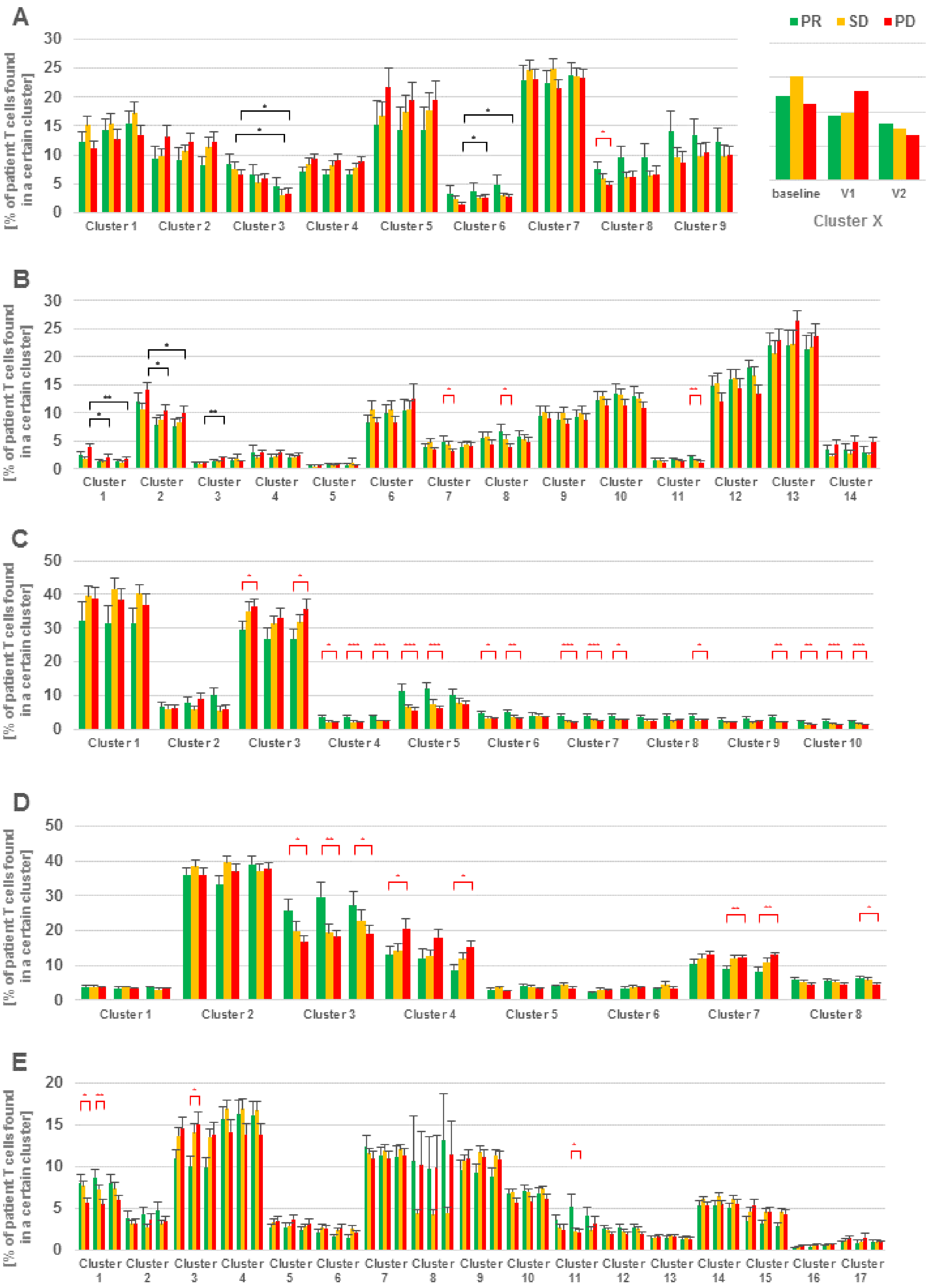

Supplementary Figure 4

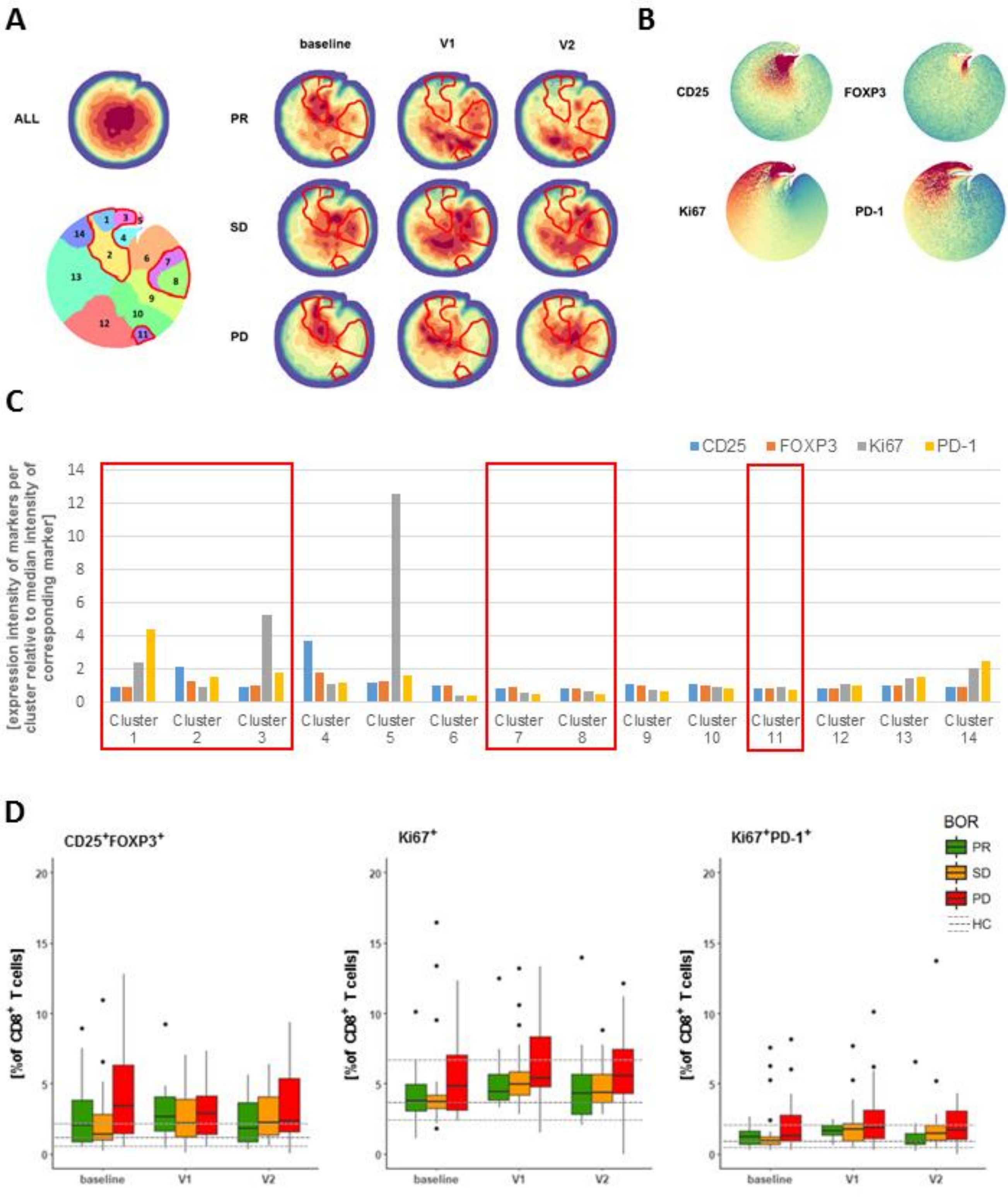

Supplementary Figure 5

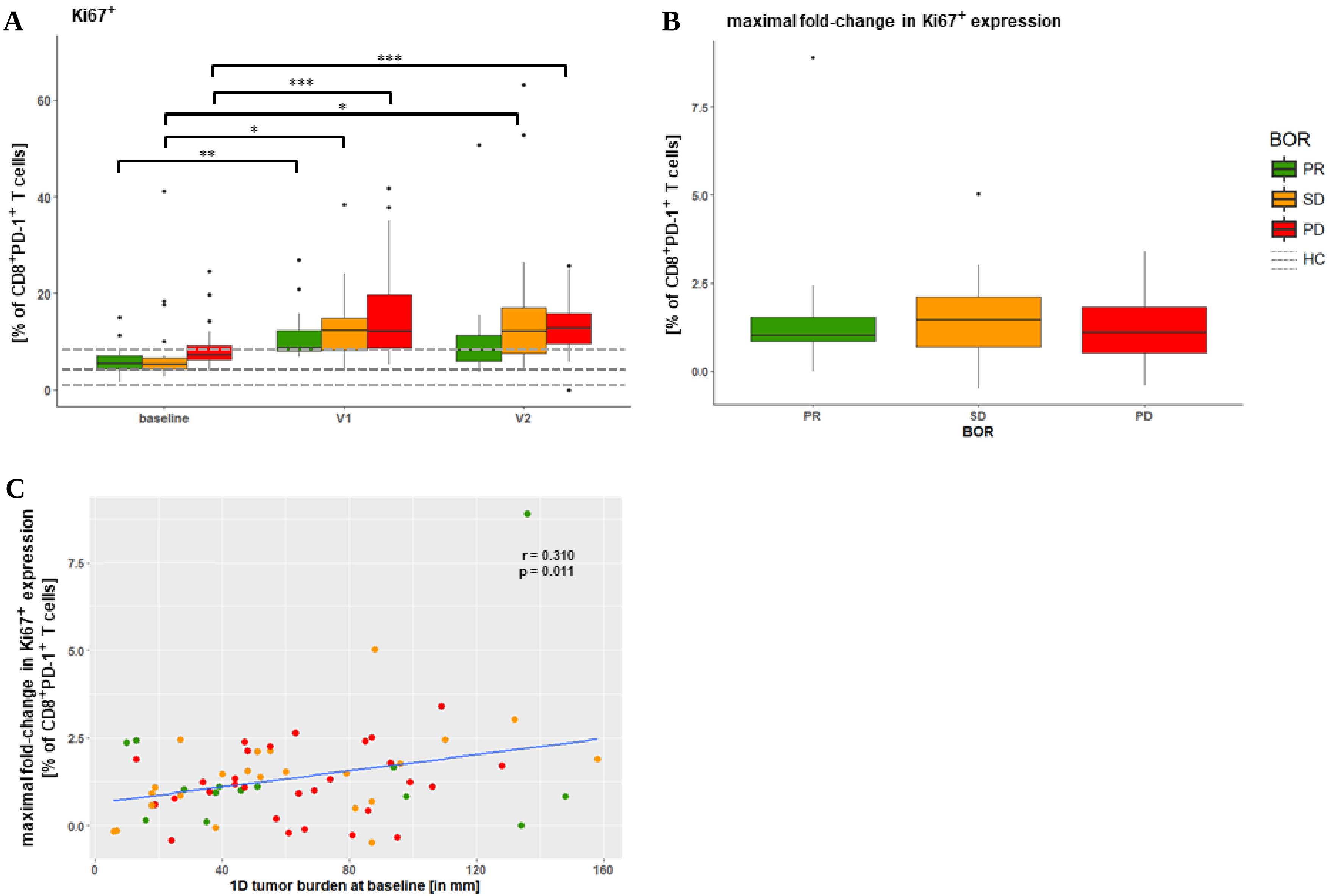

Supplementary Figure 6

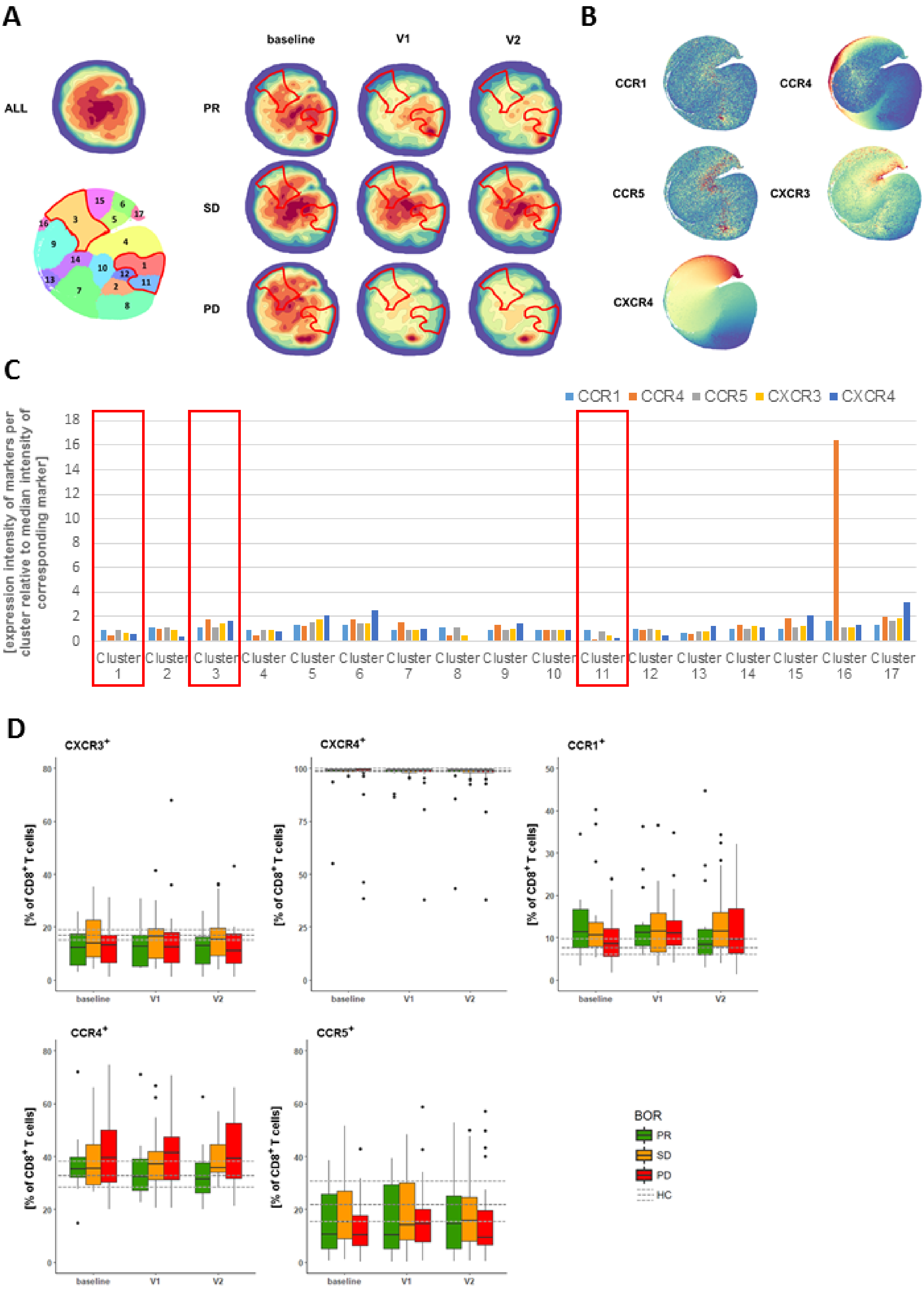

Supplementary Figure 7

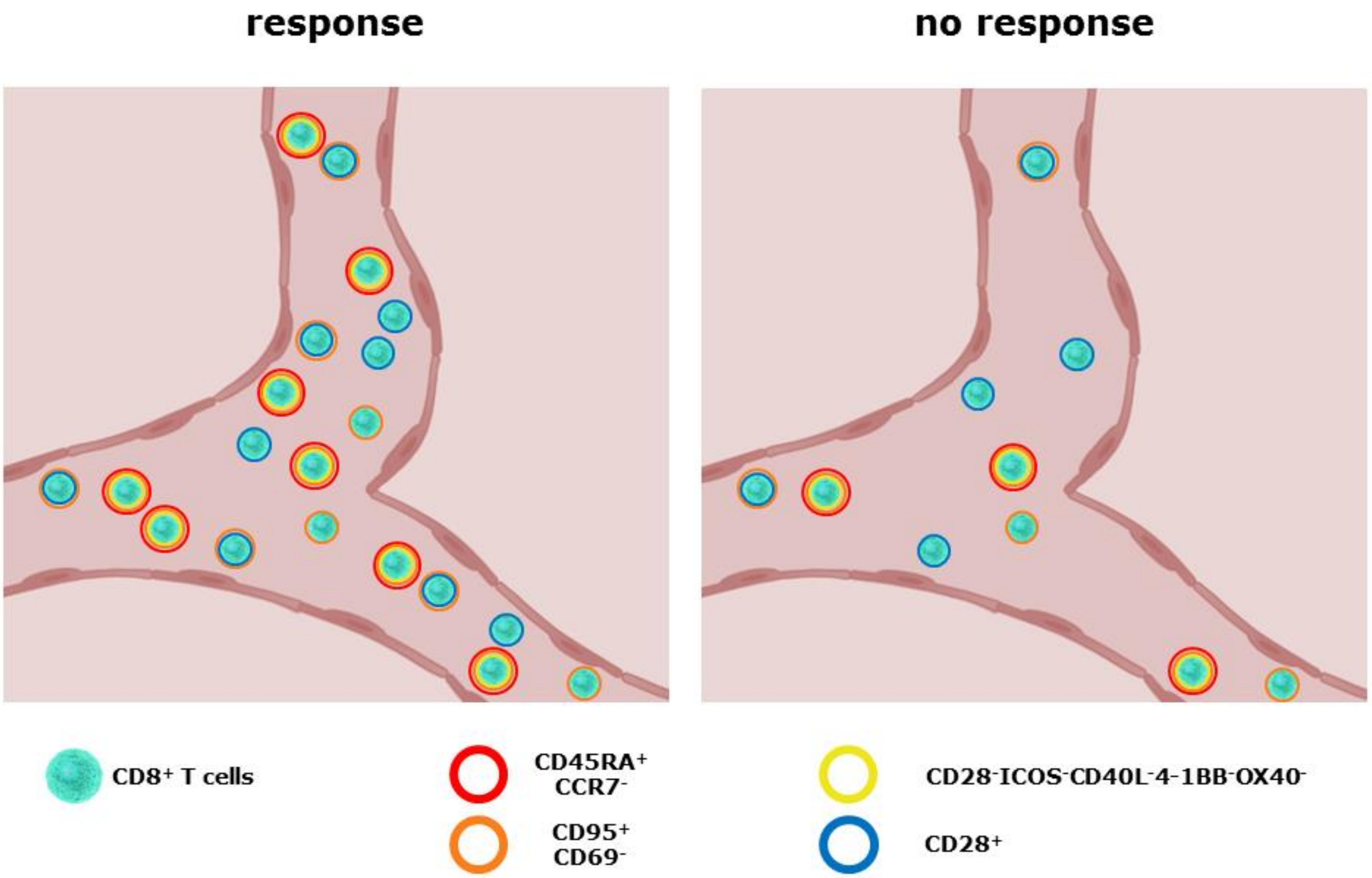

Supplementary Table I - patient characteristics

Tumor type:		NSCLC	
Treatment[a]:		Nivolumab, Q2W, 3mg/kg	
Median age (years, range):		65 (35-79)	
Sex: - female		30 (42.3%)	
- male		41 (57.7%)	
WHO performance status:	0	16 (22.5%)	
	1	37 (52.1%)	
	unknown	18 (25.4%)	
Histology of primary lung tumor:	adenocarcinoma	48 (67.6%)	
	squamous cell carcinoma	21 (29.6%)	
	great cell carcinoma	2 (2.8%)	
BOR: - progressive disease (PD)		32 (45.1%)[b]	15 (46.9%)[c]
- stable disease (SD)		25 (35.2%)	10 (31.2%)
- partial response (PR)		14 (19.7%)	7 (21.9%)
Median follow-up (days, range):		242 (35-544)	

[a] all patients received platinum-containing pre-treatment

[b] all patients enrolled in this study (71 in total) underwent staining for 28 T cell markers in frozen PBMC samples

[c] 32 of these 71 patients underwent enumeration of immune cell populations in freshly obtained blood samples

Supplementary Table II - multiplex flow cytometry panels

		markers		markers
**1**[a]	**granulocytes**	$CD45^{+}$ SSC	**eosinophils**	$CD15^{+}CD16^{-}$
			**mature neutrophils**	$CD15^{high}CD16^{high}$
			**immature neutrophils**	$CD15^{+}CD16^{+}$
	**monocytes**	$CD45^{+}$ SSC	**classical monocytes**	$CD14^{+}CD16^{-}$
			**intermediate monocytes**	$CD14^{+}CD16^{+}$
			**non-classical monocytes**	$CD14^{-}CD16^{+}$
			**DCs**	$CD14^{-}CD16^{-}CD11c^{+}$
			**M-MDSCs**	$CD14^{+}CD16^{-}CD11b^{+}HLA\text{-}DR^{low}$
	**lymphocytes**	$CD45^{+}$ SSC	**B cells**	$CD3^{-}$ $CD19^{+}$
			**NK cells**	$CD3^{-}$ $CD56^{+}$ $CD16^{+/-}$
			**T cells**	$CD3^{+}$
			**αβ - T cells**	$CD3^{+}$ $TCR\alpha\beta^{+}CD4^{+}$ or $CD8^{+}$
			**γδ - T cells**	$CD3^{+}$ $TCR\gamma\delta^{+}$

**2**[b]	T cell maturation markers	CCR7(CD197), CD45RA, CD95, CD69, CD27, CD103
**3**[b]	T cell proliferation/ regulatory T cell markers	Ki67, CD25, FOXP3, PD-1(CD279)
**4**[b]	T cell co-inhibitory receptors	CD57, LAG3(CD223), BTLA(CD272), PD-1(CD279), TIM3(CD366)
**5**[b]	T cell co-stimulatory receptors	CD28, OX40(CD134), 4-1BB(CD137), CD40L, ICOS(CD278)
**6**[b]	T cell chemoattractant receptors	CXCR3(CD183), CXCR4(CD184), CCR1(CD191), CCR4(CD194), CCR5(CD195)

[a] enumeration of immune cell populations in fresh blood samples

[b] assessment of T cell subset frequencies in PBMC samples

Supplementary Table III – analysis work scheme

**clinical parameters**	best overall response (SD/PR/PD)[a]
**time points**	baseline, V1 (2 weeks after baseline), V2 (4 weeks after baseline)
**change**	Δbaseline-V1, Δbaseline-V2, Δmax[b]

**Order of analysis**				
**1**	**2**	**3**	**4**	**5**
**immune cell populations (panel 1)**	**tSNE cluster analysis**	**defined CD4/CD8 T cells subsets (panels 2, 3)**	**CD4/CD8 T cells subsets according to single and combination of markers (panels 4-6[c])**	**CD4/CD8 T cells subsets according to accumulation of markers (panels 4-6[c])**

[a] see Methods for details on response assessment

[b] defined as maximal change from baseline measurement

[c] testing of specific marker combinations was guided by significance and was only conducted when markers yielded $p<0.05$ comparing all three BOR groups (Kruskal-Wallis).
